# Knowledge, Attitudes, and Behaviors towards Proper Nutrition and Lifestyles in Italian Diabetic Patients during the COVID-19 Pandemic

**DOI:** 10.3390/ijerph191811212

**Published:** 2022-09-07

**Authors:** Antonella Arghittu, Giovanna Deiana, Elena Castiglia, Adolfo Pacifico, Patrizia Brizzi, Andrea Cossu, Paolo Castiglia, Marco Dettori

**Affiliations:** 1 Department of Medicine, Surgery and Pharmacy, University of Sassari, 07100 Sassari, Italy; 2 University Hospital of Sassari, 07100 Sassari, Italy; 3 Department of Biomedical Sciences, University of Sassari, 07100 Sassari, Italy; 4 Ambulatorio Dietoterapia e Nutrizione Clinica, Struttura di Diabetologia e Malattie Metaboliche, ASL1, 07100 Sassari, Italy

**Keywords:** diabetes, diabetic patients, lifestyles, proper nutrition, self-management education

## Abstract

Prevention of diabetes mellitus is mainly based on a healthy lifestyle. The lockdown measures imposed during the COVID-19 pandemic resulted in major changes in daily life and social behavior, which may have an influence on diabetes self-management and glycemic control. The present work aims to assess the relationship between diabetic patients’ knowledge, attitudes, and behaviors towards proper nutrition and lifestyles in order to plan strategies for educational intervention from a health literacy perspective. Attitudes, behaviors, and knowledge of diabetic patients attending the Diabetes and Metabolic Diseases Department of the Local Health Authority of Sassari (ASL1-SS) were assessed with a cognitive survey conducted from April to July 2022. Three hundred twenty-one questionnaires were administered during the survey period. Fifty-two percent of diabetic patients were female and 48% male, with a mean age of 61.1 ± 18.5 years and 62.0 ± 15.1 years, respectively. The overall level of knowledge about the role of food and proper nutrition with respect to the risk of diabetes and its complications appeared to be generally unsatisfactory and inadequate. Nonetheless, females showed a significantly higher level of knowledge than males (*p* < 0.0001). Moreover, knowledge was seen to decrease according to the age of the patients (*p* = 0.035). As for the possible impact played by the COVID-19 pandemic on lifestyles, it should be noted that about 70% of the respondents stated that they had maintained a reasonable dietary standard or even improved it throughout. Thus, the study underlines the need to improve the knowledge of diabetic subjects about nutrition and, in particular, their self-management, positively influencing behaviors and attitudes.

## 1. Background

Diabetes is a chronic disease comprising a set of physiological dysfunctions and metabolic disorders secondary to defects in insulin secretion, insulin action, or both of these conditions [1,2,3,4]. According to the classification compiled in 1997 by the World Health Organization and the American Diabetes Association, four types of diabetes are recognized: (i) type I diabetes mellitus; (ii) type II diabetes mellitus); (iii) gestational diabetes mellitus; (iv) other forms of diabetes such as diabetes insipidus and specific types of diabetes due to monogenic syndromes, exocrine pancreatic, and drug-induced diseases [5,6,7,8].

In 2021, the International Diabetes Federation reported 537 million people aged 20 to 79 years with diabetes worldwide, and 6.7 million deaths directly attributable to the disease and its complications (of which 32.6% were of people under 60), and a global health expenditure of $966 billion [2,9]. Moreover, according to the World Health Organization, the number of diabetic adults is set to increase, and by 2030 there will be more than 642 million diabetics and 783 million by 2045. For this reason, diabetes has been identified as one of the five priority non-communicable diseases (NCDs) included in the Action Plan to tackle NCDs [10].

The prevalence of diabetes mellitus is also steadily increasing in Europe, with 62 million diabetics and 1.5 million deaths by 2021. This increase, in part due to an aging population, can be linked to variables such as obesity, poor diet, a sedentary lifestyle, and socioeconomic disparities [3,10,11].

In Italy, between 2017 and 2020, 4.7% of the adult population aged 18–69 years reported a diabetes diagnosis, more frequent in men and socio-economically disadvantaged groups (based on education and income). Prevalence is lower on average in the northwestern part of the country than in the south (7.0%) and the islands (6.7%) [11]. Sardinia is among the Regions with the highest incidence in Italy, having 50,000 cases of diabetes (type I and II) and more than 50 cases of type I diabetes per 100,000 inhabitants (age group 0–30 years) in 2021.

In the two years of this pandemic, the relationship between diabetes and COVID-19 has been explored in depth. Indeed, patients with type II diabetes mellitus, who are more prone to the risk of developing interstitial pneumonia, may become ill with COVID-19 and are more likely to develop severe forms of the disease requiring ICU admission [12,13,14].

There is a bidirectional relationship between COVID-19 and diabetes [15,16,17,18]: on the one hand, diabetes is associated with an increased risk of developing severe SARS-CoV-2 infection (a reliable indicator in predicting a complex clinical course); on the other hand, both new-onset diabetes and severe metabolic complications of pre-existing diabetes, including diabetic ketoacidosis and hyperosmolarity, have been observed in patients with COVID-19 [13,19]. Moreover, during the COVID-19 pandemic, many countries implemented lockdown measures which resulted in major changes in daily life and social behavior. These changes may have influenced diabetes self-management and glycemic control [20,21].

Prevention of diabetes mellitus is mainly based on a healthy lifestyle [22]. In fact, although numerous studies have shown that correct lifestyles can reduce the risk of type II diabetes by 58–60%, especially in those predisposed to developing the disease due to genetic and environmental factors (familiarity), a healthy diet and constant exercise positively affect the quality of life of patients with other forms of diabetes (type I, gestational, intermediate, and insipid) as well.

In this sense, patient self-management is recognized as a central component in disease prevention and treatment [23,24]. In this regard, it has been estimated that the patient is responsible for more than 95% of the actions related to disease management. Patients manage their diabetes on a daily basis within the context of the other objectives, priorities, health issues, family demands, and other personal concerns that make up their lives [24,25].

Since self-management involves cognitive, emotional, and behavioral self-regulation processes, for diabetes treatment to succeed, patients must be able to make informed decisions about how they will live with their condition [26,27].

Based on these premises, the present work aims to assess the relationship between diabetic patients’ knowledge, attitudes, and behaviors regarding nutrition, eating habits, and adoption of healthy lifestyles (e.g., physical activity), even during the lockdown, in order to plan strategies for the educational intervention of the diabetic patient from a health literacy perspective.

## 2. Methods

### 2.1. Study Setting

The present study did not require ethical approval for its observational design according to Italian law (Gazzetta Ufficiale no. 76 dated 31 March 2008). The cognitive survey targeted diabetic patients attending the Diabetes and Metabolic Diseases Department of the Local Health Authority of Sassari (ASL1-SS). Its organizational structure is defined by the “Regional Law 11 September 2020, No. 24,” which sanctions the healthcare competence of citizens residing in the territory of northern Sardinia. In total, over 15,000 diabetic patients affected by type I and type II diabetes mellitus attend ASL1-SS.

### 2.2. Survey Method

Between April and July 2022, in collaboration with the Nutrition Outpatient Clinic of the Diabetes Service of ASL1-SS, a self-developed paper questionnaire was administered (maintaining the anonymity of the patient) directly to diabetic patients at the time of their specialist visit at the same outpatient clinics. Patients were consecutively enrolled following a description of the study and receipt of informed consent. The average time to obtain consent was 15 minutes per patient, and the recruitment days were agreed upon as three sessions/week, with an average of about 20 subjects per week.

The questionnaire was tested, adjusted, and validated through a pilot study on a convenience sample of 40 diabetics (data not reported or included in the study).

To assess the attitudes, behaviors, and knowledge of diabetic patients towards proper nutrition and lifestyles, the questionnaire (Supplementary S1) was organized into 25 questions (Q1–Q25) and 4 areas of investigation: 11 personal data questions aimed at classifying the profile of the participants (Q1–7, Q10–12, Q24); 2 questions (Q13 and Q14) related to attitudes; 2 questions (Q23 and Q25) about behaviors, and 10 questions (Q8, Q9, Q15–22) about knowledge. 

### 2.3. Statistical Analysis

Data were entered into Excel (Microsoft Office, Microsoft Corporation, Redmond, WS, USA) and analyzed using STATA16 statistical software (StatCorp., Austin, TX, USA). Qualitative variables were described with absolute and relative frequencies. Associations between categorical variables were tested with chi-square. Differences between proportions were tested with the z-test. Quantitative variables were represented by measures of position and variability. Differences between means were tested with the Student’s *t*-test distribution for independent samples.

An inferential multiple regression analysis was performed in order to demonstrate the link between the outcome (knowledge levels) and individual variables (gender, age, disease duration, clinical and nutritional follow-ups, and disease self-management education). The dependent variable was constructed with a quantitative score by assigning a positive unit value for each correct item of each knowledge-related multiple-choice question. In contrast, each incorrect item was assigned a negative unit value.

The resulting variable ranged from −6 to +10, with a mean value of 3.7 and SD of 3.4. A first-type error level of 5.0% was established for inferential analyses.

## 3. Results

### 3.1. Personal Data

During the period from April 2022 to July 2022, a total of 321 questionnaires (100% compliance) were administered to a number of diabetic patients treated at the Diabetes Service of ASL1-SS.

Fifty-two percent were female and 48% male, with a mean age of 61.1 ± 18.5 years and 62.0 ± 15.1 years, respectively. No significant differences between the two genders were observed. Descriptive analysis by sex, age, weight, height, and BMI of the analyzed sample is shown in Table 1**.**

Table 2 shows the distribution of the sample based on the classification of weight status versus BMI value. Overall, about one-third of the subjects were of normal weight and 70.0% overweight. Males showed a greater tendency to be overweight, although the differences between genders were not statistically significant.

Of the 321 patients, 18 were attending their first diabetes consultation and 156 their first nutrition interview.

In addition, 310 interview subjects of the 321 to whom questionnaires were administered provided information regarding the type of diabetes diagnosed. Specifically, of these, 204 (97 females and 107 males) had type II diabetes; 106 (63 females and 43 males) had type I diabetes.

Overall, 150 subjects reported hypertension, 29 and 73 declared dyslipidemia, and obesity, respectively, and 135 did not declare any of the three comorbidities.

Regarding the percentage distribution by type of diabetes of the described coexisting conditions, 79.2% of patients with hypertension had type II diabetes; 20.8% had type I diabetes. As for dyslipidemias, 77.8% were present in patients with type II diabetes, while 22.2% were in patients with type I diabetes. In relation to dyslipidemias, these were more present in female subjects for both types of diabetes. In addition, 78.1% of patients with obesity had type II diabetes, and 21.9% had type I diabetes.

Table 3 shows the distribution of patients based on time elapsed from diagnosis of diabetes to response to the questionnaire.

Overall, most patients (79.0%) had been aware of their condition for more than two years, and only 8.0% for less than one year.

Of the respondents, 88.1% (Q12) report having received information regarding how to self-manage their disease. Of these, 89% assert that they received this information from their diabetologist, 17.0% from their General Practitioner, 7.0% from relatives and/or friends, 5.0% from the Internet, and 3.0% from nurses. In addition, 84.5% asserted that they received such information from only one person, mainly the diabetologist.

Table 4 shows the comparison between the perception of one’s weight status (obesity) as reported in the questionnaire (Q5) versus the subject’s actual status according to the classification by BMI.

Data analysis showed a prevalence of obese subjects of 29% (IC 95% = 24–34%), with a sensitivity of perceived true weight status of 67.7% (IC 95% = 63–73%) among the obese and a specificity of 95.6% (IC 95% = 93–98%). In addition, the positive predictive value (PPP) was 86.3% (IC 95% = 83.0–90.0%), as well as the negative predictive value (NPV) of 87.9% (IC 95% = 84.0–92.0%).

Taking into account individuals who reported a change in their weight during the lockdown, for 57.9%, their weight remained constant, while for 23.4%, their weight increased (1 to 20 kg, median = 4), and for 10.9%, weight decreased (2 to 30 kg, median = 5) (Q24).

### 3.2. Attitudes

The answers given to Questions Q13 and Q14, aimed at describing respondents’ attitudes about their information needs, show that 37% felt that the information was very comprehensive, 57% fairly comprehensive, and 6% little or not at all comprehensive. In contrast, among those who had received the information only from relatives/friends or the Internet, 43% considered this fairly comprehensive, and 57% little or not at all comprehensive.

**Figure 1:**
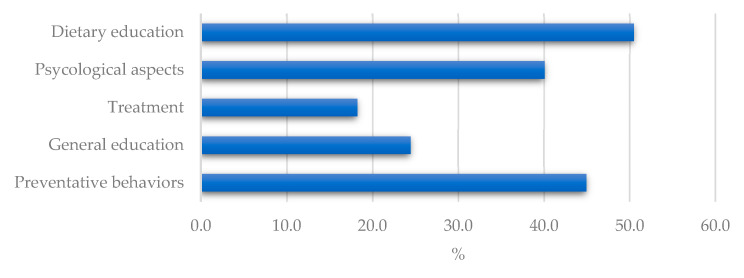
Unmet information requirements.

Figure 1 shows the information areas that patients feel have not been adequately addressed by their source.

In detail, among respondents, 50.5% would like to have received more dietary education; 45.0% more information about preventive behaviors; 40.1% information regarding the psychological effects of the disease; 24.4% felt education, in general, was lacking; and 18.2% would like to have had more information about the treatment of the condition.

### 3.3. Knowledge

Regarding the specific knowledge shown by respondents, 211 out of 256 respondents (82.4%) showed that they knew the definition of diabetic disease (Q8).

Regarding the knowledge of foods that can increase blood glucose levels (Q15), 94.2% of respondents were aware of the effect of sweets. In comparison, 90% were unaware that legumes, being a source of carbohydrates, can also lead to an increase in blood glucose. In addition, 37.6% of respondents did not believe that fruit juices increased glycemic levels, nor did mashed potatoes (52.1%). Furthermore, 30.2% and 15.4% of subjects indicated butter and red meat, respectively, were among the foods capable of raising blood sugar levels.

In relation to Q16, that is, which foods of equal weight contain more sugar, the results are reported in Table 5.

As shown in Table 5, females, compared with males, are more aware that, on an equal weight basis, fruit is the food with the highest concentration of sugars among those considered (*p* = 0.005).

Among the respondents,34.6% were aware that 50 grams of bread could be replaced with 150 grams of potatoes (Q17), with females tending to respond better, and 46.8% of males (compared with 34.7% of females) even asserting that the equivalent of 50 grams of bread is 100 grams of rice (*p* = 0.028).

In addition, 70.7% were aware that a diabetic person might consume carbohydrate-containing foods in their diet, albeit to a lesser extent than a non-diabetic person (Q18). Furthermore, 30% of respondents were unaware of the role of plant fibers in regulating the intestinal absorption of carbohydrates and lipids (Q19).

In relation to knowledge of fiber-containing foods (Q20), the results are shown in Table 6.

Specifically, 82.9% of respondents know that fiber is mostly contained in foods such as fruits, legumes, bread, and whole-wheat pasta, with a statistically significant difference between females and males (*p* = 0.002).

Moreover, 76.3% were aware of the importance of a low-salt and low-carbohydrate diet (Q21) and that a person can be properly nourished without radically changing their habits but by eliminating only the wrong behaviors (Q22).

Considering the individual level of knowledge about the investigated foods, measured through a new variable constructed from all questions asking about nutritional and dietary knowledge, Table 7 reports the multiple regression analysis of this new quantitative variable with respect to the independent variables (gender, specifically being female, age, attending diabetes follow-ups, attending nutritional consultations, years since diagnosis, education about diabetes management).

Table 7 shows that females have significantly higher levels of knowledge about food and nutrition than males and that knowledge decreases according to the age of the patient. It is also evident that following clinical and nutritional, and in particular diabetic, follow-ups significantly improve these levels of knowledge, while, paradoxically, having received self-management educational interventions seems to worsen, even if not significantly, the patient’s level of knowledge.

### 3.4. Behaviors and the Pandemic

Regarding the impact of the COVID-19 pandemic on respondents’ eating habits and lifestyles, 57.6% stated that they had maintained a healthy and balanced diet, with 4.7% reporting that they had improved their diet, compared with about 30.0% of the subjects reporting that the quality of their diet had worsened (increased consumption of carbohydrates and fat) (Q23).

The propensity for physical activity during the COVID-19 emergency (Q25) is shown in Table 8.

Table 8 shows that during the COVID-19 emergency, 43.3% of the sample did not engage in, nor had they previously engaged in, physical activity, with a statistically significant difference between females (least likely) and males (*p* < 0.0001). In contrast, 26.8% reported that they maintained the levels of physical activity they regularly performed even before the pandemic, with a statistically significant difference between males (more likely) and females (*p* < 0.0001). On the other hand, 16.5% of respondents had ceased activity, 2.8% had increased activity levels, and 4.1% had begun exercising, with no significant differences between genders.

Taking into account individuals who reported a change in their weight during the lockdown (Q24), Table 9 reports the distribution of weight changes in relation to the self-reported levels of physical activity.

Table 9 shows that an average weight gain of about 3 kg was seen in all those subjects who did not engage in physical activity or stopped engaging in physical activity during the lockdown. Conversely, a weight loss was observed in subjects who maintained or increased their level of physical exercise during the pandemic, with mean reduction values of just over 2 kg and 4.5 kg, respectively. However, those who reported starting to exercise during the emergency period experienced an increase in weight which must be considered in conjunction with dietary changes. 

## 4. Discussion

The survey made it possible to detect the knowledge, attitudes, and some behaviors of a set of 321 diabetic patients, well represented by gender and type of diabetes, who are referred to the nutrition outpatient clinic of the Diabetes Service of ASL1-SS, Sassari, during the period April 2022–July 2022.

Overall, as shown in Table 1, the sample is homogeneous in terms of age in the two sexes, with an average age of just over 60. With regard to anthropometric parameters, average excess weight in both genders should be noted, higher in males, albeit not significantly (BMI 28.3 ± 5.2 vs. 27.2 ± 5.9). Specifically, stratification by weight class (Table 2) showed that over 60% of females exceed normal weight, with 34% overweight and 28% obese, values that in males are over 70%, with 43.1% overweight and 28% obese. Thus, obesity appears to be highly prevalent but homogeneously represented in both genders. This result is in line with findings in the literature [25,28,29].

By analyzing Table 3, it appears noteworthy that about one-third of obese subjects did not perceive their overweight status. Conversely, just over 4% of the non-obese subjects (accounting for about 8% of the total overweight subjects) believed themselves to be obese. This is a phenomenon already known in the nutritional field, also described for children when parents do not perceive their child’s excess weight status, even in cases of obesity, but rather attribute such a florid state to a healthy condition and thus fail to implement the appropriate educational and behavioral interventions for proper nutrition and correct lifestyles aimed at maintaining/achieving the correct weight [30,31,32,33,34,35,36].

In adults, this phenomenon may be compounded by a psychological aspect of denying one’s condition, considering obesity as a morbid state in its own right, highlighting even further the need for appropriate educational interventions aimed at changing people’s attitudes regarding this major determinant of health [37,38]. In this regard, when analyzing the various comorbidities reported by the sample of diabetic individuals, slightly less than half (47%) of the subjects suffered from hypertension, 9% from dyslipidemia, and as many as 22% from obesity. Indeed, the latter state, when measured by BMI, brought said value as high as 29%. 

Moreover, out of the total number of diabetic subjects, 58% showed at least one of the three co-morbidities investigated (Hypertension, Dyslipidemia, and Obesity), 18% showed at least two, and 2% showed three. This high prevalence of comorbidities, in line with what has been reported in the literature, supports the need for interventions to correct the diet and lifestyles of these patients in view of the strong interplay of the comorbidities themselves to increase the risk of serious complications and severe outcomes [39,40,41].

Regarding knowledge about diabetic disease, foods, and nutrition, worrying results emerged from the survey, only partially reflecting what has also been reported in the literature. These results highlighted that the patients’ need for information was not being met. In particular, about one-fifth of patients did not know the etiopathogenesis of the condition they have. In addition, the overall level of knowledge about the role of food and proper nutrition (Questions 15 to 22) with respect to the risk of diabetes and its complications appeared to be generally unsatisfactory and inadequate for the purposes of proper management of one’s dietary behaviors or for making correct health choices [42,43].

Multiple regression analysis in this context showed that females have significantly higher levels of knowledge about food and nutrition than males, that knowledge decreases in line with patient age, and that receiving clinical and nutritional. In particular diabetological follow-ups significantly improve these levels of knowledge. Paradoxically, undergoing self-management educational programs seems to worsen, albeit not significantly, patients’ knowledge levels [44,45,46]. This is thought-provoking and, only in part, can be attributed to the fact that several patients stated that they received information exclusively from relatives, friends, or via the Internet [47,48,49,50,51]. Certainly, increased age, as shown in the regression analysis, may have played a major role in limiting the understanding of the information received. Still, in any case, the patients appeared to be aware that they needed further and more in-depth information, particularly regarding dietary education, prevention, and the psychological aspects related to the disease [52,53,54].

As for the possible impact played by the COVID-19 pandemic on lifestyles, while the literature is rich in studies on the effects of infection on diabetes exacerbation and vice versa on the effect of diabetes as a determinant for more severe COVID-19 disease outcomes, the same cannot be said regarding the effect of lockdown on nutrition and the hypothesized reduction in physical activity [13,16,24,55,56]. In fact, although one might think that a lockdown must necessarily reduce physical activity levels while increasing calorie intake, in fact, a relevant part of the population was able to take advantage of the lockdown to pay more attention to proper nutrition at home as opposed to for example, consuming frugal and unbalanced meals during work breaks outside the home in non-pandemic times. Conversely, others suffered greatly from social distancing and home isolation by compensating with food for their discomfort, indulging in sedentary living, and sometimes interrupting the daily physical activity they had previously partaken in [57,58,59,60].

Overall, the main limitation of this study is that the questionnaire is self-developed and not standardized. Moreover, the sample size was quite small; despite this, the results obtained confirm the presence of some aspects of knowledge, attitudes, and behaviors, that partially emerged also in other territorial contexts in diabetic subjects. In particular, during the lockdown period, the extreme variability of behavior may have been critical for diabetic patients. This study also sought to analyze this aspect. Although the level of response was not satisfactory due to often conflicting results, nevertheless, it should be noted that about 70% of the respondents stated that they had maintained a reasonable dietary standard or even improved it. It is also observed that about 58% managed to maintain their weight, which is not necessarily positive considering the excess weight noted and discussed above. With regard to the level of weight changes of those whose weight changed, it seems appropriate to point out that weight loss occurred in those individuals who maintained or increased their level of physical activity during the pandemic. However, those who reported starting to exercise during the lockdown went through an increase in weight—an aspect to be considered alongside dietary changes [59,60].

## 5. Conclusions

Our study has the merit of highlighting the knowledge, attitudes, and behaviors of diabetic patients, not only describing the effects of the pandemic on diabetes exacerbation and vice versa but also investigating the effect of lockdown on nutrition and the hypothesized reduction in physical activity. Consequently, the study underlines the need to improve the knowledge of diabetic subjects about nutrition and, in particular, their self-management, positively influencing behaviors and attitudes. Although most of the patients attend specialized centers, a very high percentage (over 10%) received information only from unreliable sources. These findings are even more worrying, considering that the patients were recruited from a diabetology center. It, therefore, emerges that unreliable sources of information, both within the family and on the Internet, can be decisive in hindering the adoption of correct lifestyles. 

In conclusion, the results of this study underline important margins for educational initiatives geared toward diabetic patients in our healthcare field. So much still needs to be done regarding accurate knowledge of the disease, nutrition, and correct eating behaviors in conjunction with adequate physical activity. Such initiatives will have to be tailored to the target audience, particularly taking into account the determinants shown to be significantly associated with information needs, attitudes, and behaviors, such as gender, age, and experience, in addition, of course, to the patient’s clinical conditions and co-morbidities. Thus, health communication is one of the cross-sectoral areas of communication destined to assume a central and strategic role in institution-citizen relationships.

## Figures and Tables

**Table 1:** Distribution by gender of personal data recorded.

Variables	Females	Males	Total
Mean Age (Years)	61.1	62.0	61.5
(min–max; SD)	(14–93; ±18.5)	(17–92; ±15.1)	(14–93; ±16.9)
Mean Weight (kg)	70.0	84.2	76.8
(min–max; SD)	(40–125; ±15.4)	(50–160; ±16.9)	(40–160; ±176)
Mean Height (cm)	160.4	172.4	1662
(min–max; SD)	(140–175; ±6.8)	(144–190; ±6.7)	(140–190; ±9.0)
Mean BMI	27.2	28.3	27.7
(min–max; SD)	(16–54.9; ±5.9)	(18.4–46.7; ±5.2)	(16–54.9; ±5.6)

**Table 2:** Distribution of the sample by gender and by classification based on BMI.

BMI Classification	No.	%	Females	%	Males	%
underweight	7	2.2	6	3.6	1	0.6
normal weight	92	28.7	58	34.7	34	20.4
overweight	129	40.2	57	34.1	72	43.1
mild obesity	58	18.1	29	17.4	29	17.4
moderate obesity	27	8.4	14	8.4	13	7.8
severe obesity	8	2.5	3	1.8	5	3.0
Total	321	100.0	167	100.0	154	100.0

**Table 3:** Distribution of patients by gender and time since diagnosis.

Time Elapsed Since Diagnosis	No.	%	Females	%	Males	%
>2 years	229	71.3	115	68.8	114	74.0
2 years	13	4.0	6	3.6	7	4.6
1 year	24	7.5	12	7.2	12	7.8
<1 year	24	7.5	13	7.8	11	7.1
Non-respondent	31	9.7	21	12.6	10	6.5
Total	321	100.0	167	100.0	154	100.0

**Table 4:** Comparison between perception of one’s weight status and actual classification by BMI.

	Obese (BMI ≥ 30)	Not Obese (BMI < 30)	Total
Obesity Perception	63	10	73
Non-Obesity Perception	30	218	248
Total	93	228	321

**Table 5:** Knowledge, by gender, of foods based on sugar content.

Foods Believed to Be High in Sugar	No.	%	Females	%	Males	%
Meat	2	0.6	2	1.2	0	0.0
Cheese	25	7.8	8	4.8	17	11.0
Fruit	143	44.6	87	52.1 *	56	36.4 *
Pasta	123	38.3	56	33.5	67	43.5
Don’t know	28	8.7	14	8.4	14	9.1
Total	321	100.0	167	100.0	154	100.0

* statistically significant difference (*p* = 0.005).

**Table 6:** Knowledge of fiber-containing foods.

Which Foods Contain Fiber?	No.	%	Females	%	Males	%
Meat/fish	20	6.2	6	3.6	14	9.1
Olive oil	4	1.3	2	1.2	2	1.3
Fruits/legumes/bread/whole grain Pasta	266	82.9	149	89.2 *	117	76.0 *
Don’t know	31	9.7	10	6.0	21	13.6
Total	321	100.0	167	100.0	154	100.0

* Statistically significant difference (*p* = 0.002).

**Table 7:** Multiple regression analysis by level of food knowledge and independent variables.

Knowledge Level	Coef.	Std. Err.	*t*	P > |t|	[95% Conf Interval]	
Gender (Female)	1.391866	0.3629023	3.84	0.0000	0.677	2.106
Age	−0.0231557	0.010915	−2.12	0.035	−0.044	−0.002
Diabetes Follow-up	2.692909	0.8508683	3.16	0.002	1.018	4.368
Nutritional consultation	1.203884	0.4077708	2.95	0.003	0.401	2.007
Years since diagnosis	0.1542569	0.212719	0.73	0.469	−0.265	0.573
Diabetes management Education	−1.071152	0.6401288	−1.67	0.095	−2.331	0.189
Constant	−0.7051361	1.607398	−0.44	0.661	−3.870	2.460

**Table 8:** Physical exercise during the pandemic.

Did You Engage In Physical Activity?	No.	%	Females	%	Males	%
No, but I didn’t exercise before either	139	43.3	90	53.9 *	49	31.8 *
No, I stopped exercising	53	16.5	32	19.2	22	14.3
Yes, I maintained regular physical activity	86	26.8	28	16.8 *	58	37.7 *
Yes, I increased my physical activity	9	2.8	5	3.0	4	2.6
Yes, I started exercising	13	4.1	5	3.0	8	5.2
No answer	21	6.5	7	4.2	13	8.4
Total	321	100.0	167	100.0	154	100.0

* Statistically significant difference (*p* < 0.0001).

**Table 9:** Mean and median values of weight changes in relation to self-reported physical activity levels during the pandemic.

Did You Exercise during the COVID-19 Lockdown? Did You Engage in Physical Activity?		Weight Changes	
	No.	Mean kg	Median kg
No, but I didn’t exercise before either	41	3.12	4
No, I stopped exercising	27	3.07	3
Yes, I maintained regular exercise	19	−2.11	−3
Yes, I exercised more	5	−4.60	−5
Yes, I started exercising	4	8.75	4.5
Total	96		

## Data Availability

The data presented in this study are available on reasonable request from the corresponding author.

## Supplementary Materials

The following supporting information can be downloaded at: https://www.mdpi.com/article/10.3390/ijerph191811212/s1, Supplementary S1: Questionnaire.
