# Supplementary material for: Knowledge, Attitudes, and Behaviors towards Proper Nutrition and Lifestyles in Italian Diabetic Patients during the COVID-19 Pandemic
Source: Int J Environ Res Public Health. 2022 Sep 7;19(18):11212. doi: 10.3390/ijerph191811212 (PMC9517272; doi:10.3390/ijerph191811212)
Supplement: Supplementary file 1 [file ijerph-19-11212-s001.zip › ijerph-1884893-supplementary.pdf]

Supplementary S1. QUESTIONNAIRE

Q1. Gender Woman Man

Q2. Age ____________ years

Q3. Weight _____________ kg

Q4. Height _____________ cm

Q5. Do you suffer from any of the diseases listed? (indicate one or more answers)

o High blood pressure

o Dyslipidaemia

o Obesity

o No answer

Q6. Is this your first visit to a diabetologist? (indicate one answer)

o Yes

o No

Q7. Have you ever had a nutritional consultation? (indicate one answer)

o Yes

o No

Q8. What is diabetes? (indicate one answer)

o A chronic disease characterized by a relative increase in insulin

o A chronic disease characterized by high blood glucose levels

o A defect in metabolism due to massive ingestion of sweets

o All of the above

Q9. What type of diabetes do you have? (indicate one answer)

o Type 2 non-insulin-dependent diabetes

o Type 1 insulin-dependent diabetes

o Don't know

Q10. How long have you known that you have diabetes? (please indicate an answer)

o More than 2 years

o For about 2 years

o For about 1 year

o For less than 6 months

o No answer

Q11. Have you been informed about the self-management of the condition? (indicate one answer)

o Yes

o No

o No answer

Q12. If yes, by whom? (indicate one or more answers)

o General Practitioner

o Nurse

o Diabetologist

o Relatives

o Internet

Q13. Was the information provided comprehensive?

o Very

o Quite

o A little

o Not at all

Q14. What do you think are the least treated aspects? (indicate one or more answers)

o Prevention

o Education

o Treatment

o Psychological aspects

o Dietary education

o All of the above

Q15. In your opinion, which of these foods could influence the rise in blood sugar levels? (indicate one or more answers)

o Butter

o White meat

o Fruit juices

o Extra virgin olive oil

o Mashed potatoes

o Legumes

o Red meat

o Sweets

Q16. Which of these foods, by weight, contains more sugar? (indicate one answer)

o Meat

o Cheese

o Fruit

o Pasta

o Don't know

Q17. Imagine that your diet includes 50g of bread for lunch. Which of the following replacements is correct? (indicate one answer)

o 150g potatoes

o 100g rice

o 50g mozzarella cheese or 2 eggs

Q18. Can a diabetic eat pasta, rice, legumes? (indicate one answer)

o Yes, but in very small quantities

o Yes in the same percentage as non-diabetics

o Meat and fish are preferable in order to better control blood sugar

o Don't know

Q19. Why is vegetable fiber useful in the diet of diabetics? (indicate one answer)

o Because they are rich in vitamins

o They reduce intestinal absorption of carbohydrates and fats

o Don't know

Q20. In which foods is fiber found? (indicate one answer)

o Meat and fish

o Olive oil

o Fruit, legumes, bread and wholemeal pasta

o Don't know

Q21. It is important that the diabetic's diet be: (indicate one answer)

o Low in salt and carbohydrates

o Low in spices and plain

o Usable by the rest of the family

o Don't know

Q22. In order to follow a diet, the diabetic: (indicate one answer)

o Must completely change his or her eating habits

o Can learn to eat adequately while maintaining most of his or her eating habits

o Don't know

Q23. During the COVID emergency period your diet (indicate one answer)

o Remained healthy and balanced

o Improved compared to the previous period

o Worsened with increased consumption of carbohydrates

o Worsened with increased fat consumption

Q24. During the COVID emergency period your weight (indicate one answer)

o Remained stable

o Increased (indicate the increase in kg _______)

o Decreased (indicate the decrease in kg ________)

Q25. During the COVID emergency period did you engage in physical exercise (indicate an answer)

o No, but I did not exercise before either

o No, I stopped exercising

o Yes, I maintained regular physical activity

o Yes, I increased physical activity compared before

o Yes, I started exercising, whereas before I did not
